# Diversity of endophytic bacterial and fungal microbiota associated with the medicinal lichen *Usnea longissima* at high altitudes

**DOI:** 10.3389/fmicb.2022.958917

**Published:** 2022-09-02

**Authors:** Qi Wang, Jun Li, Jie Yang, Yue Zou, Xin-Qing Zhao

**Affiliations:** ^1^State Key Laboratory of Microbial Metabolism, Joint International Research Laboratory of Metabolic and Developmental Sciences, School of Life Sciences and Biotechnology, Shanghai Jiao Tong University, Shanghai, China; ^2^R&D Center, JALA Group Co., Ltd., Shanghai, China

**Keywords:** *Usnea longissima*, lichen microbiota, endophytic microorganism, PacBio amplicon sequencing, high altitudes

## Abstract

Endophytic microbial communities of lichen are emerging as novel microbial resources and for exploration of potential biotechnological applications. Here, we focused on a medicinal lichen *Usnea longissima*, and investigated its bacterial and fungal endophytes. Using PacBio 16S rRNA and ITS amplicon sequencing, we explored the diversity and composition of endophytic bacteria and fungi in *U. longissima* collected from Tibet at five altitudes ranging from 2,989 to 4,048 m. A total of 6 phyla, 12 classes, 44 genera, and 13 species of the bacterial community have been identified in *U. longissima*. Most members belong to Alphaproteobacteria (42.59%), Betaproteobacteria (33.84%), Clostridia (13.59%), Acidobacteria (7%), and Bacilli (1.69%). As for the fungal community, excluding the obligate fungus sequences, we identified 2 phyla, 15 classes, 65 genera, and 19 species. Lichen-related fungi of *U. longissima* mainly came from Ascomycota (95%), Basidiomycota (2.69%), and unidentified phyla (2.5%). The presence of the sequences that have not been characterized before suggests the novelty of the microbiota. Of particular interest is the detection of sequences related to lactic acid bacteria and budding yeast. In addition, the possible existence of harmful bacteria was also discussed. To our best knowledge, this is the first relatively detailed study on the endophytic microbiota associated with *U. longissima*. The results here provide the basis for further exploration of the microbial diversity in lichen and promote biotechnological applications of lichen-associated microbial strains.

## Introduction

Lichens are widely distributed in a variety of terrestrial habitats and contribute significantly to global mineral cycling (Knops et al., 1991). Continuous interests have been focused on the evolution of lichen symbioses as well as community ecology (Erlacher et al., 2014; Spribille et al., 2022). Lichens belong to special symbionts formed by obligate fungi and algae or cyanobacteria through mutualism (Noh et al., 2021). Inside lichens, other microorganisms, such as endophytic bacteria and fungi, have been shown to be additional components of lichen micro-ecosystems (Diederich, 2003; Piercey-Normore, 2010). Since lichens inhabit various stressful environments such as Antarctica or dry deserts (Yu et al., 2018), it is of great interest to explore specialized microbes accommodated in lichen. Studies on the diversity of the lichen microbiome may contribute to not only a deeper understanding of the function of the microbial communities but also further exploration of lichen-derived microbial strains for potential applications (Yu et al., 2018; Prateeksha et al., 2019; Shamsun et al., 2019; Sargsyan et al., 2021; Shishido et al., 2021).

It is widely accepted that endophytes, as ubiquitous colonizers of organisms, can play an indispensable role in regulating host growth and development, such as increased yield and phytoremediation (Saikkonen et al., 2004; Yu et al., 2018). However, the importance of endophytes in lichens has long been ignored. Until the 1930s, studies on endophytes of lichens, especially bacteria, were reported, and it was generally believed that lichen bacteria played the role of nitrogen-fixing bacteria in lichen communities (Ladha et al., 1997; Piercey-Normore, 2010). Recent studies suggested that endophytes associated with lichens can produce many bioactive secondary metabolites, such as alkaloids, steroids, and peptides (Spribille et al., 2016; Suryanarayanan and Thirunavukkarasu, 2017). Additionally, increasing studies have discovered that endophytic microorganisms associated with lichen can produce a variety of unique and effective bioactivities, indicating their potential for biomedical applications (Prateeksha et al., 2019; Dawoud et al., 2020).

Previous studies on endophytes mainly focused on identifying culturable microorganisms to reveal their role in the environmental process (Alexander, 1978; Suryanarayanan et al., 2017). However, due to the heterogeneity of culture media and culture conditions, most microorganisms remain unculturable (Martiny, 2020; Atanasov et al., 2021). Fortunately, with rapid advances in high throughput sequencing technology, we can obtain more information about microbial communities through analysis of the amplicon or metagenomic sequences (Kumar et al., 2021; Wang et al., 2021b). For instance, previous studies have initially explored lichen-related bacterial communities using high-throughput sequencing of 16S ribosomal RNA gene (16S rRNA) and metagenomic analyses (Grube and Berg, 2009; Hodkinson and Lutzoni, 2009; Swamy and Gayathri, 2021). Roche 454 pyrosequencing of internal transcription spacer (ITS) was also used to assess the diversity and distribution of endophytic fungal communities associated with seven lichen species from Svalbard, showing high diversity of fungi (Zhang et al., 2015). However, so far, studies on endolichenic microbiota mainly focused on either bacterial or fungal communities. It is necessary to fully explore the entire endophytic microbial diversity for a comprehensive understanding of the lichen microbiome.

*Usnea longissima*, living in areas with clean air in China, is a medicinal lichen that has been widely used to relieve pain, control fever, and treat ulcers (Wang et al., 2021a). As a traditional Chinese medicinal lichen with high medicinal value, *U. longissima* can produce a lot of secondary metabolites which have strong antibacterial and anti-inflammatory effects, including barbatic acid, diffractaic acid, and usnic acid (Changlei et al., 2016). An endophytic fungus from *U. longissima* was proved to inhibit quorum sensing and biofilm formation of the pathogenic bacterium *Pseudomonas aeruginosa* (Prateeksha et al., 2019), suggesting that endophytic microorganisms may have potential medical and biotechnological applications. Therefore, it is of great significance to study the endophytic microbial communities of *U. longissima*. Although a preliminary study has explored the microbes of *U. longissima* from Yunnan, China (He and Zhang, 2012), the samples investigated were limited, and only symbiotic fungi and algae were discussed, whereas no information about the endophytic microbial community has been reported. As a result, until now, the endophytes in this important lichen remain a mystery, which requires further investigation.

In this study, we collected five samples of *U. longissima* at different altitudes in Tibet to jointly explore the endophytic bacterial and fungal communities as well as their unique characteristics, providing the basis for their potential applications. By 16S rRNA and ITS amplicon sequencing, we explored the following aspects of the microbiota through diversity analysis: (1) the community diversity and structural differences of endophytic bacteria and fungi at different altitudes; (2) the dominant community within *U. longissima*; and (3) endophytic microorganisms of *U. longissima* related to potential application. To our best knowledge, this is the first report on the relatively detailed analysis of microbiota in *U. longissima*. Our results shed new light on the microbial diversity of lichens and may be helpful for further understanding the environmental adaptation and evolution of lichens at high altitude regions.

## Materials and methods

### Experimental materials

The samples in this study were collected at different altitudes of Nyingchi in Tibet Autonomous Region, Southwest China. Nyingchi is located in the middle reaches of the Yarlung Zangbo River, with an average altitude of 3,100 m. We collected five samples of *U. longissima* in December 2021. Specifically, we sampled at altitudes of 2,989 m (U1), 3,383 m (U2), 3,676 m (U3), 3,680 m (U4), and 4,048 m (U5) above sea level on the mountain of Nyingchi (Table 1). We took multiple individuals from each sampling site as one sample, and three duplicate samples were, respectively, taken in the five groups (5 altitudes × 3 replicates). All samples were collected and placed in sterilized bags and immediately stored in incubators with dry ice. All samples were rinsed in sterile water, bathed in 75% ethanol for 30 s, then 3.75% NaClO for 1 min, and later rinsed three times with sterile water. Finally, 100 μl of the washed water was taken and inoculated in LB and YPD (Yeast Extract Peptone Dextrose Agar) for 7 days to verify the surface sterilization efficiency (Matsumoto et al., 2021).

**Table 1:** Information on sources of the samples used in this study.

Sample name	Longitude	Latitude	Height/m	Source tree
U1	94°45′26′ E	29°50′32′ N	2,989	*Picea asperata*
U2	94°43′54′ E	29°43′01′ N	3,383	*Abies georgei*
U3	94°24′22′ E	29°45′42′ N	3,676	*Sorbus pohuashanensis*
U4	94°24′24′ E	29°45′42′ N	3,680	*Platycladus orientalis*
U5	94°25′11′ E	29°48′18′ N	4,048	*Rhododendron* spp.

### Assessment of microbial community using PacBio sequencing

Total community DNA of the endophytic bacteria and fungi from the samples was extracted by the OMEGA Soil DNA Kit (M5635-02) according to the manufacturer’s protocol (Omega Bio-Tek, Norcross, GA, United States). NanoDrop ND-1000 spectrophotometer (Thermo Scientific, Wilmington, CA, United States) was used to measure the total DNA concentration and purity. Furthermore, DNA quality was verified with 1.2% agarose gel electrophoresis.

The complete sequence of 16S RNA and ITS genes were amplified by the PacBio platform, which has the advantage of providing more accurate species-level information. Due to the particularity of experimental materials, most of the invalid host ITS sequences of the obligate fungus (belonging to the *Dolichousnea* genus) were mixed in total amplified sequence by PacBio sequencing. Therefore, we combined amplicon information of the ITS1 region obtained by Illumina sequencing to jointly reveal more fungal community diversity. The primers used were listed in Supplementary Table S1.

The 25 μl PCR reactions were conducted by the following parameters, 5 μl of Q5 reaction buffer (5×), 5 μl of Q5 High-Fidelity GC buffer (5×), 0.25 μl of Q5 High-Fidelity DNA Polymerase (5 U/μl), 2 μl (2.5 mM) of dNTPs, 1 μl (10 μM) of each forward and reverse primer, 2 μl of DNA template, and 8.75 μl of ddH_2_O. The resulting PCR products were detected by 2% agarose gel electrophoresis and purified with Agencourt AMPure Beads (Beckman Coulter, Indianapolis, IN), and quantified using the PicoGreen dsDNA Assay Kit (Invitrogen, Carlsbad, CA, United States). In addition, negative control without the addition of any template was performed and no bands were detected, indicating that there was no contamination in the amplification process.

### Bioinformatic analysis of the fungal and bacterial sequences

The original off-machine data sequenced by PacBio was processed by circular consensus sequencing (CCS) to obtain the original FASTQ sequence. At the same time, the reverse complementary sequence was transposed to the forward sequence according to primer information. The sequence denoising and the acquirement of amplicon sequence variants (ASVs) used the classify-sklearn naïve Bayes taxonomy classifier in the feature-classifier plugin for classification and referred to the NT database (Bokulich et al., 2018). The quality of the original sequences was screened by Fastp, and the sequences with a mass fraction below 20 were removed according to the reads tail base. Next, a sliding window of 50 bp was set on reads, and reads less than 50 bp after quality control were removed when the average quality value was <20 (Matsumoto et al., 2021). In addition, sequences containing uncertain nucleotides (N) were deleted. Bar codes with more than 2 base mismatches are not allowed. The 16S rRNA of Cyanobacteria, Mitochondria, and Chloroplast, as well as ITS sequences of the host of *U. longissima* was deleted from the bacterial and fungal datasets, respectively. UCHIME was used for the removal of Chimeric sequences (Edgar et al., 2011). ASV represents different microbial classifications in a marker gene-based microbiome data set at the highest resolution available (100% similarity).

### Statistical analysis

*Quantitative Insights Into Microbial Ecology* 2 (QIIME2) was used to calculate the alpha diversity of endophytic bacterial and fungal communities (numbers of ASVs). The index of Shannon (species diversity), Chao1 (species richness), and Species Coverage were calculated to estimate the abundance, diversity, and coverage of endophytic microbial communities in *U. longissima* at different altitudes. A total of 999 permutations and combinations were evaluated using QIIME2 pair-up Adonis test and variance analysis to obtain differences in beta diversity. The principal coordinate analysis (PCoA) was visualized based on the classified Bray Curtis distance matrix (Sorensen, 1948). Venn diagrams were also drawn in R packages, presenting common and unique ASVs among different samples to represent the similarities and differences of groups at different altitudes. Due to the intergroup uniqueness of fungi, no common ASV was found between groups except for obligate fungus sequences. Therefore, we tried to explore the interaction between bacterial groups in *U. longissima* through the correlation analysis. The complexity of endophytic bacteria communities was evaluated by co-occurrence network analysis based on SparCC method. Gephi (version 0.9.2) was used to estimate topological properties, degree distribution, and modular analysis of network graphs. Nodes in the constructed network represent ASVs of different groups, while edges represent significant positive or negative correlations between groups. In order to further explore the bacterial community information in *U. longissima*, we used the full-length 16S rRNA sequence of ASVs to show the position of each ASV in the evolutionary tree and the evolutionary distance between each other. Non-singleton ASVs were aligned with mafft and used to construct a phylogeny with fasttree2 (Katoh et al., 2002; Callahan et al., 2016).

## Results

### The alpha diversity of endophytic microbial communities in *Usnea longissima*

**Figure 1:**
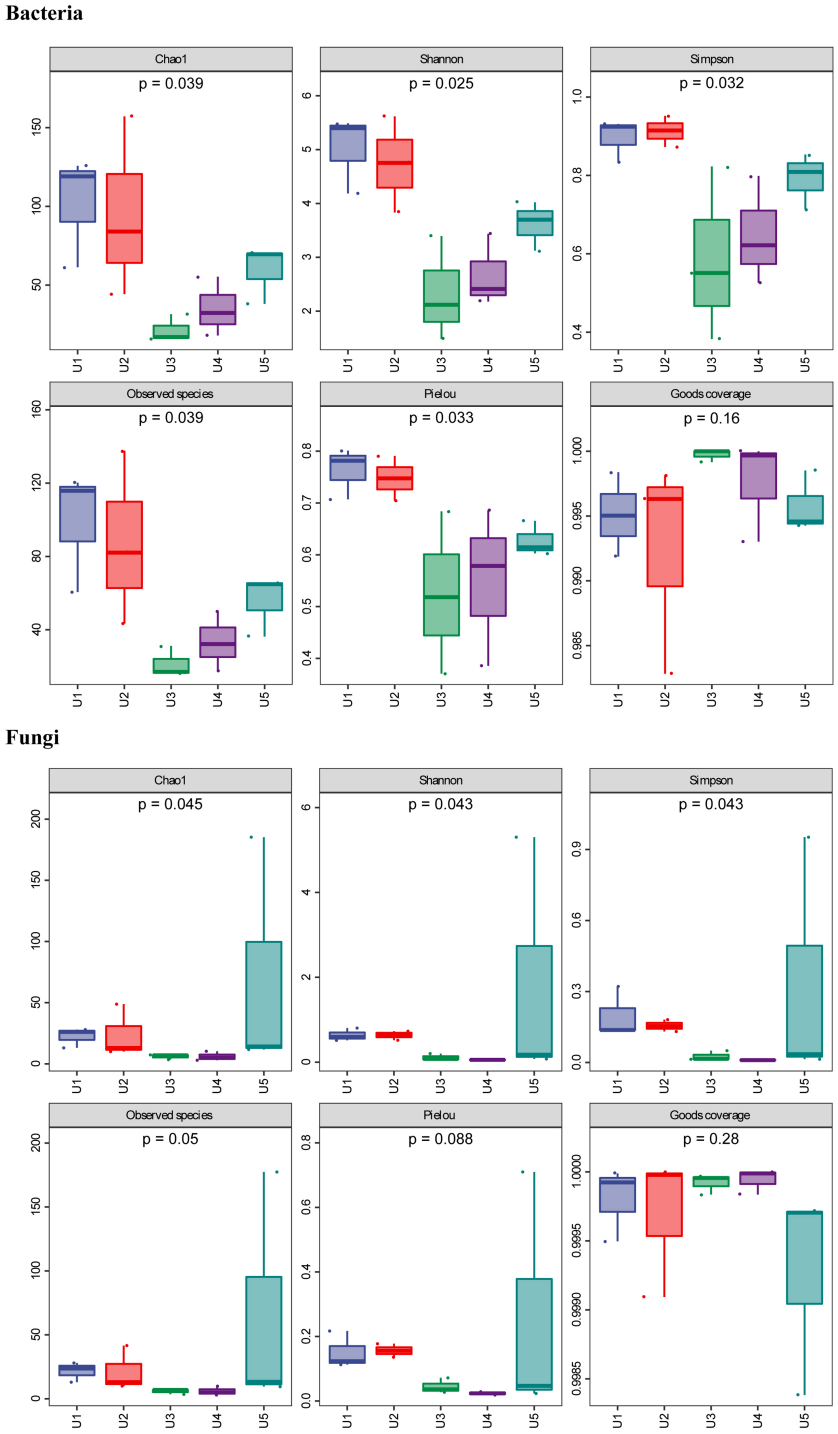
Alpha diversity of bacterial and fungal communities associated with *Usnea longissima*. The samples were collected from five different altitudes. The value of Chao1 (species richness), Simpson (species diversity), Shannon (species diversity), and Pielou (species evenness) index were counted. Observed species and Goods coverage were also calculated and presented. The number under the diversity index label was the *p*-value of the Kruskal—Wallis test.

In this study, we found no growth of any bacteria and fungi when testing the surface sterilization efficiency using the washed water, suggesting that the microbes we detected all belong to endophytic microbes. In alpha diversity analysis, both the Goods coverage index of bacterial and fungal communities were more than 99% (Figure 1), suggesting that our data were close to the actual community. In addition, the rarefaction curve tends to be flat, indicating that the sequencing results are sufficient to reflect the microbial diversity contained in the current five groups (Supplementary Figure S1).

In the case of the bacterial community, we found that although all samples belong to the same lichen species of *U. longissima*, the richness and diversity of the endophytic microbial community growing at different altitudes still differ to some extent. According to the Chao1 index (species richness) and the number of observed species, two *U. longissima* samples, U1 and U2 from relatively low altitudes had higher ASV richness than other groups (Figure 1). Nevertheless, U5 from the highest altitude had a moderate ASV richness and was higher than the U3 and U4 groups which are from the middle altitudes. Based on the Simpson (species diversity), Shannon (species diversity), and Pielou (species evenness) index, it was found that compared with U3 and U4, U1, U2, and U5 had higher species diversity.

In the PacBio fungal sequencing results, the alpha diversity index of U5, Shannon and Simpson indexes, was higher than that of the other four groups, indicating that U5 had a relatively unique community and richness (Figure 1). In the remaining four groups, we found that although abundant obligate fungus sequences were detected in all the samples, there were still some differences in the endophytic microbial communities between groups. For instance, U1 and U2 from the low altitudes appeared to have higher Chao1 and Simpson index than that of U3 and U4 in terms of endophytic fungal communities, which was consistent with the analysis of endophytic bacteria. Similar results were obtained using the PacBio sequencing and the Illumina sequencing, for example, the endophytic fungal communities of U3 and U4 showed the lowest species diversity and richness in both sequencing results. Furthermore, the overall Chao1 index of fungal communities was lower than that of bacterial communities, except for U5 from the highest altitude. The only slight difference was observed. For example, the results of the Illumina sequencing indicate that U1 from the low altitude appeared to have the highest diversity and richness judging from the alpha index, while U5 and U2 ranked second and third, respectively (Supplementary Figure S2).

### The beta diversity of endophytic microbial communities in *Usnea longissima*

**Figure 2:**
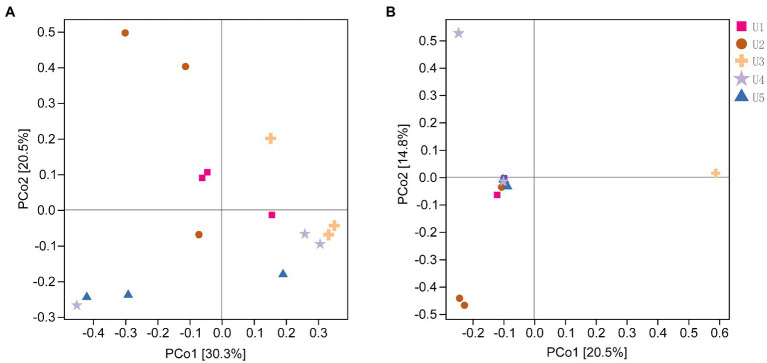
The Principal Coordinate Analysis (PCoA) of endophytic microbial communities associated with *Usnea longissima* at different altitudes by Bray-Curtis distance matrix. The distribution of the endophytic bacterial **(A)** and fungi **(B)** communities associated with *U. longissima* was shown. Each sample has three biological replicates. The value of the axis represents the percentage of variance that can be explained by each axis.

The results were supported by PERMANOVA that there was an overall difference in the structure of both bacterial communities (*p* = 0.004) and fungal communities (*p* = 0.001) in samples at different altitudes, whereas no significant difference was observed between the two groups (Supplementary Table S2). The PCoA results supported that the U2 showed a unique bacterial community structure in comparison with the other four groups, and this result was also reflected in the fungal PCoA (Figure 2). In the fungal community structure, we found that most samples by PacBio sequencing were highly concentrated, except for U2 and U3. It is worth mentioning that U3 showed the highest consistency of three parallel samples in both Illumina and PacBio sequencing (Figure 2B; Supplementary Figure S3).

### Taxonomic composition of the bacterial community

Overall, 404,813 complete 16S rRNA sequences of high-quality reads were classified into 552 ASVs with 100% sequence similarity. In the five different groups, the numbers of ASVs obtained in U1, U2, U3, U4, and U5 were 227, 206, 47, 79, and 114, respectively. Additionally, although each of the five *U. longissima* samples had a unique bacterial community structure, they also shared 11 ASVs (Supplementary Figure S4).

**Figure 3:**
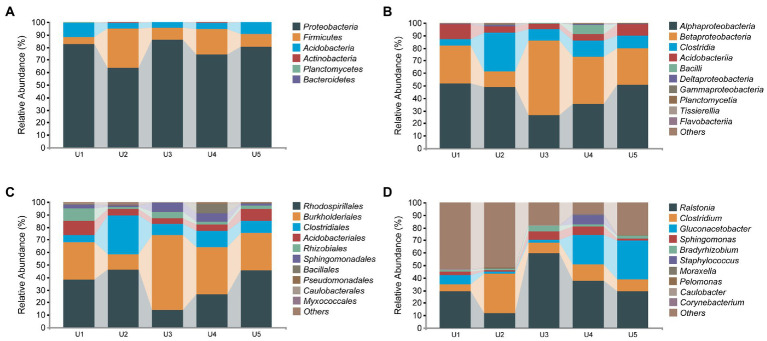
Relative abundance distribution of endophytic bacterial communities in *Usnea longissima*. **(A–D)** The relative abundance that is distributed at the phylum, class, order, and genus levels, respectively.

In the bacterial microbial community, we detected a total of 6 phyla associated with *U. longissima*, which were divided into at least 12 classes according to the classification of bacterial ASVs (Figure 3). At the phylum level, the highest relative abundance was detected in Proteobacteria (77.34%), which was reflected in all samples from five different altitudes. Subsequently, Firmicutes (15%) and Acidobacteria (7%) ranked second and third, respectively. At the class level, most sequences have been identified, with only about 0.03% to 0.4% remaining unidentified. Among these, the core microbiota consists of five classes with the relative abundance more than 1%, Alphaproteobacteria (42.59%), Betaproteobacteria (33.84%), Clostridia (13.59%), Acidobacteria (7%), and Bacilli (1.69%), respectively. In the five groups, U3 and U4 hosted a lower abundance of Alphaproteobacteria in comparison with the other samples, 26.71% and 35.5%, respectively. Moreover, in other classes, we found some differences between groups, such as Clostridia with the highest abundance in U2 and Betaproteobacteria with the highest abundance in U3 (Figure 3B). At the order level, a high abundance of Rhodospirillales, Burkholderiales, and Clostridiales was observed. At the genus level, we detected a total of 11 genera, but there were still large shares of specific sequences that were not detected in the existing database. Among the known genera, *Ralstonia* and *Clostridium* are the dominant genera presented in all five groups, followed by *Gluconacetobacter* and *Sphingomonas* which also occupy large abundance. However, some genera are specific to certain groups. For example, *Corynebacterium* and *Stenotrophomonas* only exist in U4 and U5, respectively.

We measured the complete sequence of 16S rRNA, ~1,500 bp in length. Thus, the accuracy of the species identification of endophytic microorganisms was better guaranteed compared to those previously reported using the partial sequence of 16S rRNA. The 12 species of bacteria that can be detected were roughly divided into two groups: potentially beneficial bacteria and potentially pathogenic bacteria. More specifically, the potential beneficial bacteria mainly include *Geobacter lovleyi*, *C. glutamicum*, *Propionicimonas paludicola*, *Lactobacillus helveticus*, and *Nakamurella multipartita*. Potentially pathogenic bacteria include *Clostridium perfringens*, *Staphylococcus aureus*, *Moraxella Lacunata*, *Escherichia coli*, *R. solanacearum*, *Neisseria meningitides*, and *Brevundimonas diminuta*.

### Taxonomic composition of the fungal community

**Figure 4:**
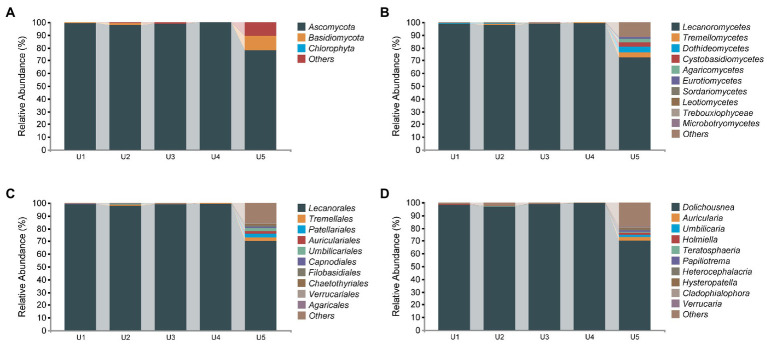
Relative abundance distribution of endophytic fungal communities in *Usnea longissima*. **(A–D)** The relative abundance that are distributed at the phylum, class, order, and genus levels, respectively.

In the PacBio sequencing results, 423,783 high-quality fungal sequences were divided into 320 ASVs based on 100% sequence similarity. Compared to that of the bacterial communities, the diversity of fungal communities we detected was relatively limited, which included Ascomycota (95%), Basidiomycota (2.69%), and unidentified phyla (2.5%) (Figure 4). Despite many ITS sequences of the obligate fungus detected in the samples (abundance ranging from 96.85% to 99.57%, except for 72.6% in U5), we can still observe some specific sequences belonging to different taxa in the samples. In total, Tremellomycetes was present in almost all samples except U3. Cystobasidiomycetes and Dothideomycetes were found in U1 and U2, respectively, whereas both were observed in U5. The remaining unidentified Fungi (10.81%), Agaricomycetes (2.49%), Eurotiomycetes (1.32%), and nine classes with an abundance of <1% were found only in the high altitude sample U5.

Although few ASVs from endophytic fungi were obtained compared with that of bacteria, we detected more genera and species. Specifically, 65 genera and 19 different species have been detected, although most of them are present in U5. The previous study compared growth of *Abies georgei* var. *smithii* at different altitudes in Tibet, and found that 4,100 m is the most suitable altitude, and abiotic stress will occur if the growth altitude is higher or lower than 4,100 m (Guo et al., 2016). We detected the most abundant endophytic fungi in U5 in this study, and we hypothesized that 4,048 m may be that close to the suitable altitude for the growth of *U. longissima*. More studies will be performed to support this hypothesis. *Auricularia cornea* (2.36%) was the most abundant species of U5 in the identified species. In addition, we also detected *Soosiella minima*, *Curvibasidium pallidicorallinum*, *Hypholoma capnoides*, *Cladophialophora chaetospira*, and budding yeast *Saccharomyces cerevisiae* as well as a few other species with low abundance (Supplementary Figure S5).

### Correlation of bacterial communities revealed from network structure

In terms of class, the sub-network showed that Alphaproteobacteria was considered the central element, forming two main lines (Supplementary Figure S6). Deltaproteobacteria Alphaproteobacteria, Acidobacteria, and Planctomycetia form a line, and there was a strong positive correlation. Furthermore, Clostridia, Alphaproteobacteria, and Betaproteobacteria, as the second line, had a negative correlation.

**Figure 5:**
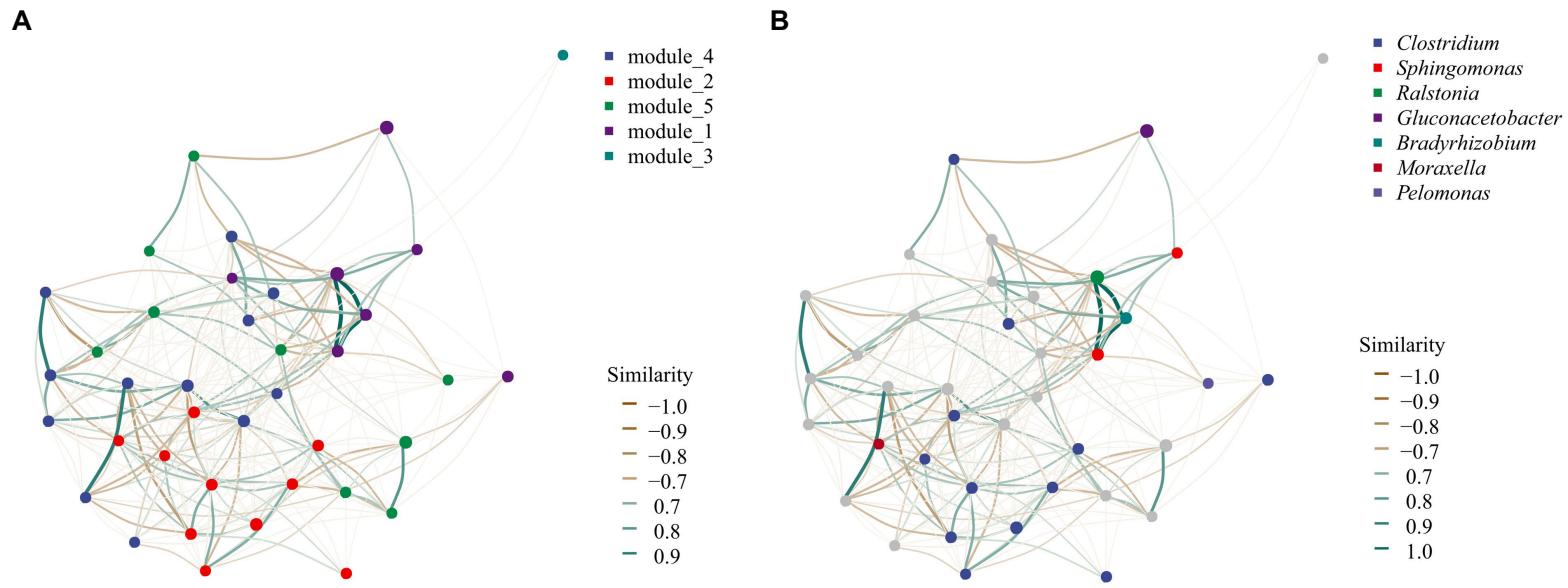
Co-occurrence network analysis of bacterial community modules and genera based on SparCC method. **(A)** The module distribution of network analysis. Five different colors of dots were used to represent the five modules, representing the possible functional groups. **(B)** Network structure at the genus level. Each point can be called a node, which can represent an ASV or a taxon in the community. The connecting line between two points can be called edge, which represents the distribution trend of positive or negative correlation between the two points connected.

The endophytic bacterial group of *U. longissima* appeared to have five putative modules which might be useful to perform specific ecological functions (Figure 5). Module 1 was mainly composed of 7 ASVs, including 3 highly correlated species, ASV_503 (*Ralstonia*), ASV_267 (*Sphingomonas*), and ASV_588 (*Bradyrhizobium*). Module 2 covers 10 ASVs, including nine strains of *Clostridium perfringens* and ASV_247 (*Moraxella lacunata*). In contrast, in module 3, only the single ASV_9 existed, which belonged to Rhizobiales and was classified as Alphaproteobacteria. In addition, 10 ASVs of Acetobacteraceae family, ASV_514 (belonging to the Rhizobiales), and ASV_181 (*C. perfringens*) of Rhizobiales constitute module 4. Most ASVs cannot be accurately identified at the species level. Our results showed that the sequences of most ASVs were unique and different from sequences known to the database. Finally, the interaction among ASV_335 (Acidobacteriaceae), 6 ASVs of Acetobacteraceae, ASV_369 (*C. perfringens*), ASV_123, and ASV_123 (*Pelomonas* genus) was shown in Module 5.

### Phylogenetic analysis of endophytic bacteria associated with *Usnea longissima*

**Figure 6:**
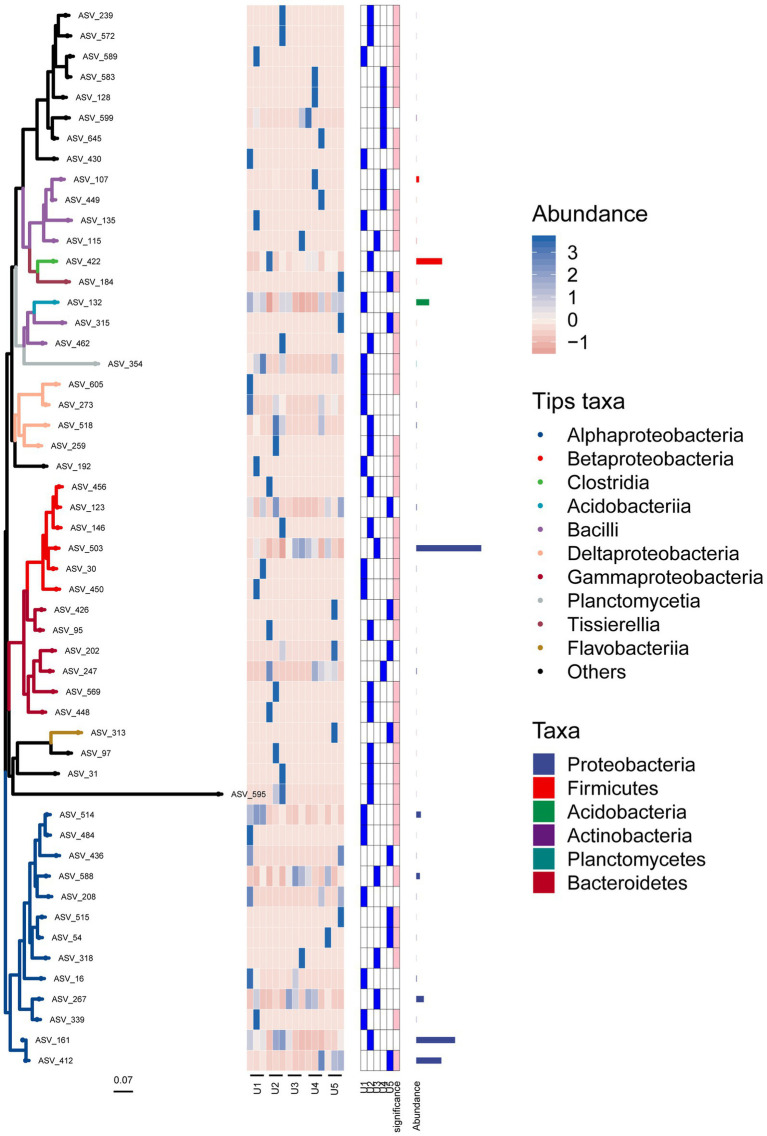
Position of each ASV associated with the endophytic bacterial community in the evolutionary tree. The heat map and bar chart reflect the composition, abundance, and taxonomic information of the bacterial community. Branches of different colors represent species of different classes.

In the phylogenetic tree, we can see that the bacterial microbial community was divided into two main topological branches (Figure 6). In the first large cluster, most ASVs of the same class were clustered on the same branch, except for a few ASVs embedded in other branches. For instance, ASVs_315 and ASV_462 were not in a cluster with other species in Bacilli (i.e., ASV_107 and ASV_149). In addition, most of the ASVs that had not been specifically classified were clustered on one clade to form a monophyletic group, except for ASV_192. It was evident that these unknown species have a closer relationship and may play a similar role in the ecosystem. The second topological clade was mainly composed of some groups of Proteobacteria phylum, including Alphaproteobacteria, Betaproteobacteria, and Gammaproteobacteria. Furthermore, ASV_97 (Flavobacteriia class) and ASV_313 formed sister groups, which then congregated with ASV_31 and ASV_595. These results indicated that these unknown sequences were more closely related to Flavobacteriia than Proteobacteria.

## Discussion

Here, we conducted the first relative detailed study on the community diversity and structure of endophytic bacteria and fungi in *U. longissima* by PacBio amplicon sequencing and Illumina sequencing. All our samples were collected in high altitude areas of Tibet, which can be further divided into three groups according to relative altitude: U1 and U2 (low altitude), U3 and U4 (middle altitude), and U5 (high altitude). We revealed the core microbiota of *U. longissima* by diversity analysis and community composition. In addition, we further discussed the potential biotechnology application and ecological risks of medicinal lichen *U. longissima*.

For the bacterial community diversity, through the alpha diversity index, we found that the richness and diversity of U1 and U2 from relatively low altitudes were higher than middle and high altitude groups. Studies have shown that the harshness of the environment increases with altitude, leading to a decrease in microbial abundance and activity (Margesin and Miteva, 2011). In the analysis of fungal diversity, although only limited endophytic fungal sequences were obtained due to the influence of obligate fungus sequences, these sequences can fully reflect the real community of samples. We found the lowest diversity of U3 and U4 from middle altitudes. Our results agree with those recently reported (Zhang et al., 2022), which found that the microbial community composition of the soil at the middle altitude showed the lowest microbial diversity. It was proposed that this may be caused by altitude or lack of nutrients in the habitat (Wang et al., 2016).

From the perspective of community composition, the scatter plot of PCoA showed that U2 exhibited a unique community composition in both bacterial and fungal communities. It has been shown that lichen species and geography are the main factors influencing highly structured and diverse microbial communities (Hodkinson et al., 2012). In our study, all the samples were from the same lichen, so the influence of lichen species can be excluded. Therefore, it is likely that U2 geographical environment causes its unique endophytic microbial community structure, such as pH, temperature, and nutrient elements (Zhang et al., 2022). Moreover, the fungal microbial community structure of U3 was also unique, although this feature was not found in the bacterial community (Figure 5). Previous studies have shown that bacterial and fungal colonization patterns were different (De Andrade et al., 2021; Wang and Pecoraro, 2021), so it is understandable that the structural characteristics of bacterial and fungal populations are inconsistent in U3.

Analysis of 16S rRNA genes in the microbiome of *U. longissima* at five altitudes revealed that most members belong to Alphaproteobacteria, Betaproteobacteria, Clostridia, Acidobacteria, and Bacilli, which form the core bacterial microbiota despite environmental fluctuations. These results are consistent with the previous results obtained in other lichens (Sierra et al., 2020). Alphaproteobacteria has been found to be a core member of lichens, playing an indispensable role in nutrient absorption and lichen growth (Cardinale et al., 2008). In particular, among Alphaproteobacteria, Rhodospirillales (Bates et al., 2011), Rhizobiales (Erlacher et al., 2014), and Sphingomonadales (Cardinale et al., 2008), which are essential for the maintenance of lichens, were distributed to varying degrees in all groups. Rhodospirillales has previously been shown to be responsible for most potassium and nitrogen metabolism and to be resistant to oxidative stress in lichens (Cernava et al., 2017). Sphingomonadales in lichens has also been studied for their potential to synthesize vitamin B12 and for its resistance to cobalt and chromium (Cernava et al., 2017). Moreover, Acetobacteraceae had a high abundance in all five samples, reaching 14.29% to 45.96% of the total sequence, which has also been found in other lichens of the genus *Usnea* (Sierra et al., 2020). Furthermore, Betaproteobacteria, one of the most important groups of Proteobacteria, had a significant abundance advantage in *U. longissima*, which has been reported to be able to adapt lichens to nutritionally poor environments (Wedin et al., 2016). Indeed, almost all the *U. longissima* in this study grow at high altitudes, where the environment is relatively extreme and harsh (Mao et al., 2021). Therefore, Betaproteobacteria may play an important role in adapting to the extreme condition in Tibet. Different from other lichen studies (Bates et al., 2011; Sierra et al., 2020), the abundance of Deltaproteobacteria and Gammaproteobacteria in our study is low, which may be caused by different host genotypes and environmental characteristics.

Our data also supports the idea that *Clostridium* (belonging to Clostridia) is universally distributed in the environment (Sarria-Guzmán et al., 2016), with a widespread distribution in *U. longissima*. However, a high abundance of *C. perfringens* was identified, which has been reported as a potential pathogen in several studies (Petit et al., 1999; Zhu et al., 2017). The abundance of *C. perfringens* ranges from 1.89% to 18.81% in most samples, except in U2_1 which reaches as high as 80.30%. Dozens of wild bharals were reported to have died after being infected with *C. perfringens* in Tibet (Zhu et al., 2017). Therefore, *C. perfringens* in the lichen samples may pose a risk of animal disease. The colonization of *C. perfringens* in *U. longissima* may be due to environmental changes that intruded into the host or may have been associated with the evolution of the host. *Ralstonia*, a genus believed to cause plant blight, has also been detected in lichens. Although many sequences of the genus *Ralstonia* were detected, except for a few sequences corresponding to *R. solanacearum*, most of the sequences were different from the existing sequences in the database and might indicate the existence of new species. For these potential new species there may be functions that are different from those of the same species. In addition, a low abundance of other potentially pathogenic bacteria genera was detected in the samples, such as *Moraxella* (Blakeway et al., 2018) and *Staphylococcus* (Rossi et al., 2020).

The findings of pathogenic bacteria in our current study are very unexpected. The results warrant further systematic studies of active pathogenic endophytic bacteria in lichen samples. However, caution should be taken to conclude that the lichens represent a threat to plants or animals. Although these species are primarily reported as pathogens, strains of the same species growing in different environments can vary widely in their genomes and functions (Studier et al., 2009). In addition, whether these microorganisms are metabolically active or not also deserves our attention. From the perspective of the host, most detected pathogens are reported to be pathogenic only to animals (Zhu et al., 2017; Rossi et al., 2020), so these microorganisms may not affect the growth and development of lichens. This is the first report on potential pathogenic bacteria in lichens. Further studies are needed to determine the activity and functional properties of the colonized in *U. longissima*.

In addition, a lot of bacteria with potential biotechnological applications were found in *U. longissima*. As a typical acid-producing lichen (Changlei et al., 2016), the acidophilic microbial taxa (Acidobacteriaceae, Acidobacteria) were also found to be prevalent in our samples with relatively high abundance (7%), indicating that *U. longissima* may be a valuable resource pool for acidophilic microorganisms. Moreover, *Gluconacetobacter*, the third most abundant genus detected in our data, was present at all five altitudes. Bacteria in *Gluconacetobacter* genus have been shown to have the potential to produce large amounts of extracellular polysaccharides (Valepyn et al., 2012). Similarly, *Sphingomonas*, a core member in *U. longissima*, have the ability to promote growth and stress tolerance in the host (Asaf et al., 2018). A recent study has shown that *Sphingomonas* are highly abundant microorganisms in lichens, almost absent from the substrate in which they grow, suggesting that the endophytes of lichens are not merely extensions of microorganisms in the substrate (Leiva et al., 2021). Furthermore, bacteria that can produce propionic acid (*Propionicimonas paludicola*) and secondary metabolites with the function of lower blood pressure (*Lactobacillus helveticus*) have also been found in *U. longissima* (Akasaka et al., 2004; Chen et al., 2014), but these bacteria have not been reported in other lichen studies. Lichen-associated low-abundance strains can play important functional roles in lichen micro-ecosystems, and the products they release have been shown to be effective antibiotics at very low concentrations (Grube and Berg, 2009). *Corynebacterium glutamicum* is commonly used as producers of amino acids and nucleotides (Lee et al., 2016). It will be interesting to further explore the special microbial strains for various biotechnological applications.

It has been suggested that endophytic fungi are incubators for the evolution of endophytes and different from fungal host organisms (Grube and Berg, 2009). Lichen-related fungi of *U. longissima* mainly came from two phyla, Ascomycota and Basidiomycota, which is consistent with the previous study (He and Zhang, 2012). In addition, it is worth noting that our results are consistent with the previous study indicating that the photobiont of *U. longissima* are derived from Chlorophyta phylum, specifically from *Trebouxia* genus (He and Zhang, 2012). At the class level, Lecanoromycetes, Dothideomycetes, Leotiomycetes, Eurotiomycetes, and Sordariomycetes from Ascomycota, and sequences of Tremellomycetes and Cystobasidiomycetes from Basidiomycota were identified, which are consistent with previous studies of Antarctic and Arctic lichens where the temperature is usually low (Park et al., 2015; Zhang et al., 2015).

Interestingly, lichen-forming fungi, such as the class Tremellomycetes, have been found to exist inside *U. longissima*, especially for U5 collected from the highest altitude. According to the latest revision of the classification of Tremellomycetes (Liu et al., 2015), the genera *Papiliotrema*, *Vishniacozyma*, and *Phenoliferia* within this class were present in our samples, which were reported to occur frequently in cold ecological environments (Coleine et al., 2018). The emergence of these groups may be related to the characteristics of their environment (Selbmann et al., 2014; Coleine et al., 2018). Moreover, Dothideomycetes, known as the lineage of extremely tolerant fungi, was detected as the third most abundant class in our sample of U5 from the high altitude, which was reported to be able to withstand stressful conditions and harsh environments in the host (Chen et al., 2019). It is interesting to further explore the specified endophytes from cold environments including Tibet.

Compared with the previous study on lichen-related fungi of *U. longissima* (He and Zhang, 2012), we obtained more genus and species information through PacBio and Illumina amplicon sequencing. *Sydowia* and *Cladosporium*, previously cultured from *U. longissima* (He and Zhang, 2012), were also present in the samples we collected. However, some genera identified in the previous study (He and Zhang, 2012), such as *Mucor*, *Hypocrea*, and *Trichoderma*, were not identified in our Tibetan samples. Differences in microbiome occurrence may be that samples from different geographical environments and seasonal climates have also been reported to cause community changes (Kang et al., 2010). Importantly, we have indeed isolated some yeast strains from the samples of *U. longissima* as endophytic fungi, including budding yeast *S. cerevisiae* (unpublished), *Curvibasidium cygneicollum,* and *C. rogersii* (Bai et al., 2022), the latter of which agree with the detection of the genus *Curvibasidium* in this study. These results encourage us to further explore the culturable strains in the near future.

In total, we detected a large number of endophytic microbial communities of *U. longissima*, including some fungi that could not be identified at the phylum level, which may be potential new species in lichen (Duarte et al., 2016). Our study provides a new perspective on microbial resources for biotechnology applications. Since many of the detected microorganisms have not been successfully isolated and cultured, subsequent culture methods can be improved, including low temperature and acid enrichment (Biosca et al., 2016). We can further reveal the metabolic activity and function of endophytes in lichens through multiple omics techniques, containing metabolomics, transcriptomics, and metaproteomics (Nazem-Bokaee et al., 2021; Ullah et al., 2021). Moreover, our samples were collected in Tibet, a typical high altitude region with obvious freezing–thawing stress, which may affect the structural changes of endophytes in lichens (Leiva et al., 2021). Therefore, it is necessary to select samples with seasonal variation to explore the dynamic endophytic microbial community of *U. longissima* in future studies.

## Conclusion

In this study, we report the first detailed investigation of the endophytic bacterial and fungal community composition in *U. longissima* from five different altitudes in Tibet. Our results suggested that the endophytic microbial communities differ in samples collected at different altitudes. The relatively high diversity of the microbiota in *U. longissima* was revealed at low and high latitudes. We detected many previously unreported sequences, suggesting the existence of many new taxa. Our results recommend cautions for potentially pathogenic bacteria. Moreover, microorganisms with biotechnological application have been revealed, such as *C. glutamicum* and *S. cerevisiae*, which are the first report from lichen. Further experiments are needed to isolate the strains from the lichen samples to better characterize their special features, pathogenicity, and advantages in the production of bioactive agents. Our results shed new light on lichen microbiota for further exploration of the microbial diversity and biotechnology applications.

## Data availability statement

The datasets presented in this study can be found in online repositories. The names of the repository/repositories and accession number(s) can be found at: https://www.ncbi.nlm.nih.gov/bioproject/PRJNA838808, https://www.ncbi.nlm.nih.gov/bioproject/PRJNA838944, and https://www.ncbi.nlm.nih.gov/bioproject/PRJNA839274.

## Author contributions

X-QZ and QW designed and conceived the experiment. QW performed the data analysis and drafted the manuscript. All authors contributed to the article and approved the submitted version.

## Funding

This work was supported by grants from the National Research Foundation of China (nos. 5151101168 and 21536006).

## Conflict of interest

JL and YZ were employed by JALA Group Co., Ltd.

The remaining authors declare that the research was conducted in the absence of any commercial or financial relationships that could be construed as a potential conflict of interest.

## Publisher’s note

All claims expressed in this article are solely those of the authors and do not necessarily represent those of their affiliated organizations, or those of the publisher, the editors and the reviewers. Any product that may be evaluated in this article, or claim that may be made by its manufacturer, is not guaranteed or endorsed by the publisher.

## References

[ref1] Akasaka H. Ueki K. Ueki A. (2004). Effects of plant residue extract and Cobalamin on growth and propionate production of *Propionicimonas paludicola* isolated from plant residue in irrigated Rice field soil. Microbes Environ. 19, 112–119. doi: 10.1264/jsme2.19.112

[ref2] Alexander M. (1978). Introduction to soil microbiology. Soil Sci. 125:331. doi: 10.1097/00010694-197805000-00012

[ref3] Asaf S. Khan A. L. Khan M. A. Al-Harrasi A. Lee I. J. (2018). Complete genome sequencing and analysis of endophytic *Sphingomonas* sp. LK11 and its potential in plant growth. 3 Biotech 8, 389–314. doi: 10.1007/s13205-018-1403-z, PMID: 30175026 PMC6111035

[ref4] Atanasov A. G. Zotchev S. B. Dirsch V. M. Supuran C. T. (2021). Natural products in drug discovery: advances and opportunities. Nat. Rev. Drug Discov. 20, 200–216. doi: 10.1038/s41573-020-00114-z, PMID: 33510482 PMC7841765

[ref5] Bai L. Zhang A. Zhu Y. T. Wang X. Q. Wang M. Y. Zhao X. Q. (2022). First isolation and identification of cold adaptive yeast Curvibasidium rogersii from Usnea lichen and genome-based studies of its biological properties. Acta Microbiol. Sin. 62, 567–578. doi: 10.13343/j.cnki.wsxb.20210220

[ref6] Bates S. T. Cropsey G. W. Caporaso J. G. Knight R. Fierer N. (2011). Bacterial communities associated with the lichen symbiosis. Appl. Environ. Microbiol. 77, 1309–1314. doi: 10.13343/j.cnki.wsxb.20210220, PMID: 21169444 PMC3067232

[ref7] Biosca E. G. Flores R. Santander R. D. Díez-Gil J. L. Barreno E. (2016). Innovative approaches using lichen enriched media to improve isolation and culturability of lichen associated bacteria. PLoS One 11:e0160328. doi: 10.1371/journal.pone.0160328, PMID: 27494030 PMC4975499

[ref8] Blakeway L. V. Tan A. Lappan R. Ariff A. Pickering J. L. PeacockC S.. (2018). *Moraxella catarrhalis* restriction–modification systems are associated with phylogenetic lineage and disease. Genome Biol. Evol. 10, 2932–2946. doi: 10.1093/gbe/evy226, PMID: 30335144 PMC6241649

[ref9] Bokulich N. A. Kaehler B. D. Rideout J. R. Dillon M. Bolyen E. Knight R.. (2018). Optimizing taxonomic classification of marker-gene amplicon sequences with QIIME 2's q2-feature-classifier plugin. Microbiome 6, 1–17. doi: 10.1186/s40168-018-0470-z, PMID: 29773078 PMC5956843

[ref10] Callahan B. J. McMurdie P. J. Rosen M. J. Han A. W. Johnson A. Holmes S. P. (2016). DADA2: high-resolution sample inference from Illumina amplicon data. Nat. Methods 13, 581–583. doi: 10.1038/nmeth.3869, PMID: 27214047 PMC4927377

[ref11] Cardinale M. Castro J. Müller H. Berg G. Grube M. (2008). *In situ* analysis of the bacterial community associated with the reindeer lichen *Cladonia arbuscula* reveals predominance of Alphaproteobacteria. FEMS Microbiol. Ecol. 66, 63–71. doi: 10.1111/j.1574-6941.2008.00546.x, PMID: 18631179

[ref12] Cernava T. Erlacher A. Aschenbrenner I. A. Krug L. Lassek C. Riedel K.. (2017). Deciphering functional diversification within the lichen microbiota by meta-omics. Microbiome 5, 82–13. doi: 10.1186/s40168-017-0303-5, PMID: 28724401 PMC5518139

[ref13] Changlei S. Feng L. Jie S. Jia L. Xiao W. (2016). Optimisation and establishment of separation conditions of organic acids from *Usnea longissima* ach. By pH-zone-refining counter-current chromatography: discussion of the eluotropic sequence. J. Chromatogr. A 1427, 96–101. doi: 10.1016/j.chroma.2015.12.016, PMID: 26686561

[ref14] Chen Y. Liu W. Xue J. Jie Y. Chen X. Shao Y.. (2014). Angiotensin-converting enzyme inhibitory activity of *Lactobacillus helveticus* strains from traditional fermented dairy foods and antihypertensive effect of fermented milk of strain H9. J. Dairy Sci. 97, 6680–6692. doi: 10.3168/jds.2014-7962, PMID: 25151888

[ref15] Chen J. Wang P. Wang C. Wang X. Sun S. (2019). Fungal community demonstrates stronger dispersal limitation and less network connectivity than bacterial community in sediments along a large river. Environ. Microbiol. 22, 832–849. doi: 10.1111/1462-2920.14795, PMID: 31469494

[ref16] Coleine C. Stajich J. E. Zucconi L. Onofri S. Pombubpa N. Egidi E.. (2018). Antarctic cryptoendolithic fungal communities are highly adapted and dominated by Lecanoromycetes and Dothideomycetes. Front. Microbiol. 9:1392. doi: 10.3389/fmicb.2018.01392, PMID: 30008702 PMC6033990

[ref17] Dawoud T. M. Alharbi N. S. Theruvinthalakal A. M. Thekkangil A. Kadaikunnan S. Khaled J. M.. (2020). Characterization and antifungal activity of the yellow pigment produced by a *bacillus* sp. DBS4 isolated from the lichen *Dirinaria agealita*. Saudi J. Biol. Sci. 27, 1403–1411. doi: 10.1016/j.sjbs.2019.11.031, PMID: 32346353 PMC7182979

[ref18] De Andrade P. A. M. de Souza A. J. Lira S. P. Assis M. A. Berlinck R. G. Andreote F. D. (2021). The bacterial and fungal communities associated with Anthurium ssp. leaves: insights into plant endemism and microbe association. Microbiol. Res. 244:126667. doi: 10.1016/j.micres.2020.126667, PMID: 33338969

[ref19] Diederich L. P. (2003). Lichenicolous fungi: interactions, evolution, and biodiversity. Bryologist 106, 80–120. doi: 10.1639/0007-2745(2003)106[0080:LFIEAB]2.0.CO;2

[ref20] Duarte A. W. F. Passarini M. R. Z. Delforno T. P. Pellizzari F. M. Cipro C. V. Z. Montone R. C.. (2016). Yeasts from macroalgae and lichens that inhabit the S outh S Hetland I slands. Antarctica. Environ. Microbiol. Rep. 8, 874–885. doi: 10.1111/1758-2229.12452, PMID: 27518570

[ref21] Edgar R. C. Haas B. J. Clemente J. C. Quince C. Knight R. (2011). UCHIME improves sensitivity and speed of chimera detection. Bioinformatics 27, 2194–2200. doi: 10.1093/bioinformatics/btr381, PMID: 21700674 PMC3150044

[ref22] Erlacher A. Cernava T. Cardinale M. Soh J. Sensen C. W. Grube M.. (2014). Rhizobiales as functional and endosymbiontic members in the lichen symbiosis of *Lobaria pulmonaria* L. Front. Microbiol. 6:53. doi: 10.3389/fmicb.2015.00053, PMID: 25713563 PMC4322706

[ref23] Grube M. Berg G. (2009). Microbial consortia of bacteria and fungi with focus on the lichen symbiosis. Fungal Biol. Rev. 23, 72–85. doi: 10.1016/j.fbr.2009.10.001

[ref24] Guo Q. Li H. Zhang W. (2016). Variations in leaf functional traits and physiological characteristics of *Abies georgei* var. *smithii* along the altitude gradient in the southeastern Tibetan plateau. J. Mt. Sci. 13, 1818–1828. doi: 10.1007/s11629-015-3715-3

[ref25] He Y. Zhang Z. (2012). Diversity of organism in the *Usnea longissima* lichen. Afr. J. Microbiol. Res. 6, 4797–4804. doi: 10.5897/AJMR12.534

[ref26] Hodkinson B. P. Gottel N. R. Schadt C. W. Lutzoni F. (2012). Photoautotrophic symbiont and geography are major factors affecting highly structured and diverse bacterial communities in the lichen microbiome. Environ. Microbiol. 14, 147–161. doi: 10.1111/j.1462-2920.2011.02560.x, PMID: 21906220

[ref27] Hodkinson B. P. Lutzoni F. (2009). A microbiotic survey of lichen-associated bacteria reveals a new lineage from the Rhizobiales. Symbiosis 49, 163–180. doi: 10.1007/s13199-009-0049-3

[ref28] Kang Y. J. Cheng J. Mei L. J. Hu J. Piao Z. Yin S. X. (2010). Multiple copies of 16S rRNA gene affect the restriction patterns and DGGE profile revealed by analysis of genome database. Microbiology 79, 655–662. doi: 10.1134/S0026261710050103 21090508

[ref29] Katoh K. Misawa K. Kuma K. I. Miyata T. (2002). MAFFT: a novel method for rapid multiple sequence alignment based on fast Fourier transform. Nucleic Acids Res. 30, 3059–3066. doi: 10.1093/nar/gkf436, PMID: 12136088 PMC135756

[ref30] Knops J. Nash I. Boucher V. L. Schlesinger W. H. (1991). Mineral cycling and epiphytic lichens: implications at the ecosystem level. Lichenologist 23, 309–321. doi: 10.1017/S0024282991000452

[ref31] Kumar M. Kumar A. Sahu K. P. Patel A. Reddy B. Sheoran N.. (2021). Deciphering core-microbiome of rice leaf endosphere: revelation by metagenomic and microbiological analysis of aromatic and non-aromatic genotypes grown in three geographical zones. Microbiol. Res. 246:126704. doi: 10.1016/j.micres.2021.126704, PMID: 33486428

[ref32] Ladha J. K. Bruijn F. D. Malik K. A. (1997). Isolation of Endophytic Bacteria From Rice and Assessment of Their Potential for Supplying Rice With Biologically Fixed Nitrogen, *Vol. 775*. Netherlands: Springer, 25–36.

[ref33] Lee J. Na Y. Kim E. Lee H. Kim P. (2016). The actinobacterium *Corynebacterium glutamicum*, an industrial workhorse. J. Microbiol. Biotechnol. 26, 807–822. doi: 10.4014/jmb.1601.01053, PMID: 26838341

[ref34] Leiva D. Fernández M. F. Acevedo J. Carú M. Grube M. Orlando J. (2021). The bacterial community of the foliose macro-lichen *Peltigera frigida* is more than a mere extension of the microbiota of the subjacent substrate. Microb. Ecol. 81, 965–976. doi: 10.1007/s00248-020-01662-y, PMID: 33404820

[ref35] Liu X. Wang Q. Göker M. Groenewald M. Kachalkin A. V. Lumbsch H. T.. (2015). Towards an integrated phylogenetic classification of the Tremellomycetes. Stud. Mycol. 81, 85–147. doi: 10.1016/j.simyco.2015.12.001, PMID: 26955199 PMC4777781

[ref36] Mao K. Wang Y. Liu J. (2021). Evolutionary origin of species diversity on the Qinghai-Tibet Plateau. J. Syst. Evol. 59, 1142–1158.

[ref37] Margesin R. Miteva V. (2011). Diversity and ecology of psychrophilic microorganisms. Res. Microbiol. 162, 346–361. doi: 10.1016/j.resmic.2010.12.004, PMID: 21187146

[ref38] Martiny A. C. (2020). The ‘1% culturability paradigm’needs to be carefully defined. ISME J. 14, 10–11. doi: 10.1038/s41396-019-0507-8, PMID: 31551529 PMC6908684

[ref39] Matsumoto H. Fan X. Wang Y. Yue W. Peter K. Jie D.. (2021). Bacterial seed endophyte shapes disease resistance in rice. Nat. Plants 7, 60–72. doi: 10.1038/s41477-020-00826-5, PMID: 33398157

[ref40] Nazem-Bokaee H. Hom E. F. Warden A. C. Mathews S. Gueidan C. (2021). Towards a systems biology approach to understanding the lichen symbiosis: opportunities and challenges of implementing network modelling. Front. Microbiol. 12:1028. doi: 10.3389/fmicb.2021.667864, PMID: 34012428 PMC8126723

[ref41] Noh H. Park Y. Hong S. G. Lee Y. M. (2021). Diversity and physiological characteristics of Antarctic lichens-associated bacteria. Microorganisms 9:607. doi: 10.3390/microorganisms9030607, PMID: 33804278 PMC8001610

[ref42] Park C. H. Kim K. M. Elvebakk A. Kim O. S. Jeong G. Hong S. G. (2015). Algal and fungal diversity in Antarctic lichens. J. Eukaryot. Microbiol. 62, 196–205. doi: 10.1111/jeu.12159, PMID: 25105247

[ref43] Wang X. Pecoraro L. (2021). Analysis of soil fungal and bacterial communities in Tianchi volcano crater. Northeast China Life. 11:280. doi: 10.3390/life11040280, PMID: 33810555 PMC8066613

[ref44] Petit L. Gibert M. Popoff M. R. (1999). *Clostridium perfringens*: toxinotype and genotype. Trends Microbiol. 7, 104–110. doi: 10.1016/S0966-842X(98)01430-9, PMID: 10203838

[ref45] Piercey-Normore M. D. (2010). The lichen-forming ascomycete Evernia mesomorpha associates with multiple genotypes of Trebouxia jamesii. New Phytol. 169, 331–344. doi: 10.1111/j.1469-8137.2005.01576.x, PMID: 16411936

[ref46] Prateeksha B. R. Yusuf M. A. Upreti D. K. Singh B. N. (2019). Endolichenic fungus, Aspergillus quandricinctus of *Usnea longissima* inhibits quorum sensing and biofilm formation of *Pseudomonas aeruginosa* PAO1. Microb. Pathog. 140:103933. doi: 10.1016/j.micpath.2019.103933, PMID: 31862392

[ref47] Rossi C. C. Pereira M. F. Giambiagi-deMarval M. (2020). Underrated staphylococcus species and their role in antimicrobial resistance spreading. Genet. Mol. Biol. 43:e20190065. doi: 10.1590/1678-4685-GMB-2019-0065, PMID: 32052827 PMC7198029

[ref48] Saikkonen K. Wäli P. Helander M. Faeth S. H. (2004). Evolution of endophyte-plant symbioses. Trends Plant Sci. 9, 275–280. doi: 10.1016/j.tplants.2004.04.005, PMID: 15165558

[ref49] Sargsyan R. Gasparyan A. Tadevosyan G. Panosyan H. (2021). Antimicrobial and antioxidant potentials of non-cytotoxic extracts of corticolous lichens sampled in Armenia. AMB Exp. 11:110. doi: 10.1186/s13568-021-01271-z, PMID: 34324070 PMC8322222

[ref50] Sarria-Guzmán Y. Chávez-Romero Y. Gómez-Acata S. Montes-Molina J. A. Morales-Salazar E. Dendooven L.. (2016). Bacterial communities associated with different *Anthurium andraeanum* L. plant tissues. Microbes Environ. 31, 321–328. doi: 10.1264/jsme2.ME16099, PMID: 27524305 PMC5017810

[ref51] Selbmann L. Turchetti B. Yurkov A. Cecchini C. Zucconi L. Isola D.. (2014). Description of Taphrina Antarctica fa sp. nov., a new anamorphic ascomycetous yeast species associated with Antarctic endolithic microbial communities and transfer of four Lalaria species in the genus *Taphrina*. Extremophiles 18, 707–721. doi: 10.1007/s00792-014-0651-z, PMID: 24893860

[ref52] Shamsun N. Min-Hye J. Jae-Seoun H. (2019). Lichen-associated bacterium, a novel bioresource of Polyhydroxyalkanoate (PHA) production and simultaneous degradation of naphthalene and Anthracene. J. Microbiol. Biotechnol. 29, 79–90. doi: 10.4014/jmb.1808.08037, PMID: 30518016

[ref53] Shishido T. K. Wahlsten M. Laine P. Rikkinen J. Lundell T. Auvinen P. (2021). Microbial communities of Cladonia lichens and their biosynthetic gene clusters potentially encoding natural products. Microorganisms 9:1347. doi: 10.3390/microorganisms9071347, PMID: 34206222 PMC8304397

[ref54] Sierra M. A. Danko D. C. Sandoval T. A. Pishchany G. Moncada B. Kolter R.. (2020). The microbiomes of seven lichen genera reveal host specificity, a reduced core community and potential as source of antimicrobials. Front. Microbiol. 11:398. doi: 10.3389/FMICB.2020.00398, PMID: 32265864 PMC7105886

[ref55] Sorensen T. A. (1948). Method of establishing groups of equal amplitude in plant sociology based on similarity of species content and its application to analyses of the vegetation on Danish commons. biologiske skrifter 5, 1–34. doi: 10.1234/12345678

[ref56] Spribille T. Resl P. Stanton D. E. Tagirdzhanova G. (2022). Evolutionary biology of lichen symbioses. New Phytol. 234, 1566–1582. doi: 10.1111/nph.18048, PMID: 35302240

[ref57] Spribille T. Tuovinen V. Resl P. Vanderpool D. Wolinski H. Aime M. C.. (2016). Basidiomycete yeasts in the cortex of ascomycete macrolichens. Science 353, 488–492. doi: 10.1126/science.aaf8287, PMID: 27445309 PMC5793994

[ref58] Studier F. W. Daegelen P. Lenski R. E. Maslov S. Kim J. F. (2009). Understanding the differences between genome sequences of Escherichia coli B strains REL606 and BL21(DE3) and comparison of the E. coli B and K-12 genomes. J. Mol. Biol. 394, 653–680. doi: 10.1016/j.jmb.2009.09.021, PMID: 19765592

[ref59] Suryanarayanan T. S. Govindarajulu M. B. Rajamani T. Tripathi M. Joshi Y. (2017). Endolichenic fungi in lichens of Champawat district, Uttarakhand, northern India. Mycol. Prog. 16, 205–211. doi: 10.1007/s11557-016-1268-7

[ref60] Suryanarayanan T. S. Thirunavukkarasu N. (2017). Endolichenic fungi: the lesser known fungal associates of lichens. Mycology 8, 189–196. doi: 10.1080/21501203.2017.1352048, PMID: 30123639 PMC6059131

[ref61] Swamy C. T. Gayathri D. (2021). High throughput sequencing study of foliose lichen-associated bacterial communities from India. Mol. Biol. Rep. 48, 2389–2397. doi: 10.1007/s11033-021-06272-6, PMID: 33735409

[ref62] Ullah J. Khanum Z. Khan I. A. Khalid A. N. Musharraf S. G. Ali A. (2021). Metaproteomics reveals the structural and functional diversity of *Dermatocarpon miniatum* (L.) W. Mann. Microbiota. Fungal Biol. 125, 32–38. doi: 10.1016/j.funbio.2020.10.001, PMID: 33317774

[ref63] Valepyn E. Berezina N. Paquot M. (2012). Optimization of production and preliminary characterization of new exopolysaccharides from *Gluconacetobacter hansenii* LMG1524. Adv. Microbiol. 02, 488–496. doi: 10.4236/aim.2012.24062

[ref64] Wang T. Shen C. Guo F. Zhao Y. Chen Y. (2021a). Characterization of a polysaccharide from the medicinal lichen, *Usnea longissima*, and its immunostimulating effect *in vivo*. Int. J. Biol. Macromol. 181, 672–682. doi: 10.1016/j.ijbiomac.2021.03.183, PMID: 33798588

[ref65] Wang Y. Zheng Y. Wang X. Wei X. Wei J. (2016). Lichen-associated fungal community in Hypogymnia hypotrypa (Parmeliaceae, Ascomycota) affected by geographic distribution and altitude. Front. Microbiol. 7:1231. doi: 10.3389/fmicb.2016.01231, PMID: 27547204 PMC4975045

[ref66] Wang Z. Zhu Y. Li N. Liu H. Liu Y. (2021b). High-throughput sequencing-based analysis of the composition and diversity of endophytic bacterial community in seeds of saline-alkali tolerant rice. Microbiol. Res. 250:126794. doi: 10.1016/j.micres.2021.126794, PMID: 34062342

[ref67] Wedin M. Maier S. Fernandez B. S. Cronholm B. Westberg M. Grube M. (2016). Microbiome change by symbiotic invasion in lichens. Environ. Microbiol. 18, 1428–1439. doi: 10.1111/1462-2920.13032, PMID: 26310431

[ref68] Yu N. H. Park S. Kim J. A. Park C. Jeong M. Oh S.. (2018). Endophytic and endolichenic fungal diversity in maritime Antarctica based on cultured material and their evolutionary position among Dikarya. Fungal Syst. Evol. 2, 263–272. doi: 10.3114/fuse.2018.02.07, PMID: 32467890 PMC7225575

[ref69] Zhang R. Tian X. Xiang Q. Penttinen P. Gu Y. (2022). Response of soil microbial community structure and function to different altitudes in arid valley in Panzhihua, China. BMC Microbiol. 22, 1–11. doi: 10.1186/s12866-022-02500-6, PMID: 35366810 PMC8976301

[ref70] Zhang T. Wei X. Zhang Y. Liu H. Yu L. (2015). Diversity and distribution of lichen-associated fungi in the Ny-Ålesund region (Svalbard, high Arctic) as revealed by 454 pyrosequencing. Sci. Rep. 5, 1–10. doi: 10.1038/srep14850, PMID: 26463847 PMC4604449

[ref71] Zhu L. Zhou W. Wang T. Xiang H. Ji X. Han Y.. (2017). Isolation of *Clostridium perfringens* type A from wild bharals (*Pseudois nayaur*) following sudden death in Tibet, China. Anaerobe 44, 20–22. doi: 10.1016/j.anaerobe.2017.01.004, PMID: 28082244

