# Supplementary material for: Diversity of endophytic bacterial and fungal microbiota associated with the medicinal lichen Usnea longissima at high altitudes
Source: Front Microbiol. 2022 Sep 2;13:958917. doi: 10.3389/fmicb.2022.958917 (PMC9479685; doi:10.3389/fmicb.2022.958917)
Supplement: Supplementary file 1 [file Table_1.DOCX]

Supplementary Material

# 1 Supplementary Figures and Tables

## Supplementary Tables

**Supplement Table S1.** The specific primer information

| **Gene** | **Forward primer** | **Reverse primer** |
| --- | --- | --- |
| 16S | 5’-AGAGTTTGATCMTGGCTCAG-3’ | 5’-ACCTTGTTACGACTT-3’ |
| ITS | 5’-CTTGGTCATTTAGAGGAAGTAA-3’ | 5’-CCGTGTTTCAAGACGGG-3’ |
| ITS1 | 5’-CTTGGTCATTTAGAGGAAGTAA-3’ | 5’-GCTGCGTTCTTCATCGATGC-3’ |

**Supplement Table S2.** Analysis on the group difference of endophytic bacteria and fungi of *Usnea longissima*

**Bacteria**

| **Group1** | **Group2** | **Samplesize** | **Permutations** | **pseudoF** | **pvalue** | **qvalue** |
| --- | --- | --- | --- | --- | --- | --- |
| all | - | 15 | 999 | 2.098867 | 0.004 | - |
| U1 | U2 | 6 | 999 | 2.31243 | 0.104 | 0.175 |
| U1 | U3 | 6 | 999 | 3.664479 | 0.105 | 0.175 |
| U1 | U4 | 6 | 999 | 1.71664 | 0.23 | 0.2875 |
| U1 | U5 | 6 | 999 | 2.896015 | 0.09 | 0.175 |
| U2 | U3 | 6 | 999 | 2.662513 | 0.088 | 0.175 |
| U2 | U4 | 6 | 999 | 1.507333 | 0.202 | 0.2875 |
| U2 | U5 | 6 | 999 | 2.290338 | 0.092 | 0.175 |
| U3 | U4 | 6 | 999 | 1.032937 | 0.303 | 0.333 |
| U3 | U5 | 6 | 999 | 3.773333 | 0.097 | 0.175 |
| U4 | U5 | 6 | 999 | 0.832973 | 0.333 | 0.333 |

**Fungi**

| **Group1** | **Group2** | **Samplesize** | **Permutations** | **pseudoF** | **pvalue** | **qvalue** |
| --- | --- | --- | --- | --- | --- | --- |
| all | - | 15 | 999 | 2.555685 | 0.001 | - |
| U1 | U2 | 6 | 999 | 1.603962 | 0.202 | 0.202 |
| U1 | U3 | 6 | 999 | 4.09574 | 0.1 | 0.123333 |
| U1 | U4 | 6 | 999 | 1.631359 | 0.111 | 0.123333 |
| U1 | U5 | 6 | 999 | 1.060686 | 0.104 | 0.123333 |
| U2 | U3 | 6 | 999 | 7.179522 | 0.102 | 0.123333 |
| U2 | U4 | 6 | 999 | 2.507204 | 0.103 | 0.123333 |
| U2 | U5 | 6 | 999 | 1.659519 | 0.093 | 0.123333 |
| U3 | U4 | 6 | 999 | 6.999451 | 0.096 | 0.123333 |
| U3 | U5 | 6 | 999 | 4.11809 | 0.087 | 0.123333 |
| U4 | U5 | 6 | 999 | 1.658487 | 0.101 | 0.123333 |

## Supplementary Figures


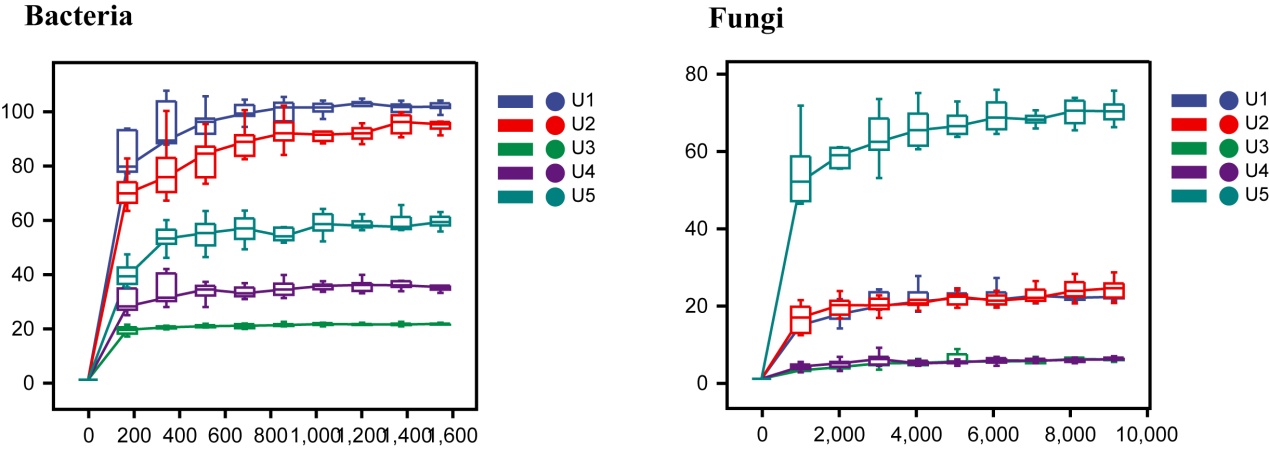


**Supplement Fig. S1.** The rarefaction curve of bacterial and fungal communities. The curve tends to be flat, indicating that the current sequence information is enough to represent the community microbes.


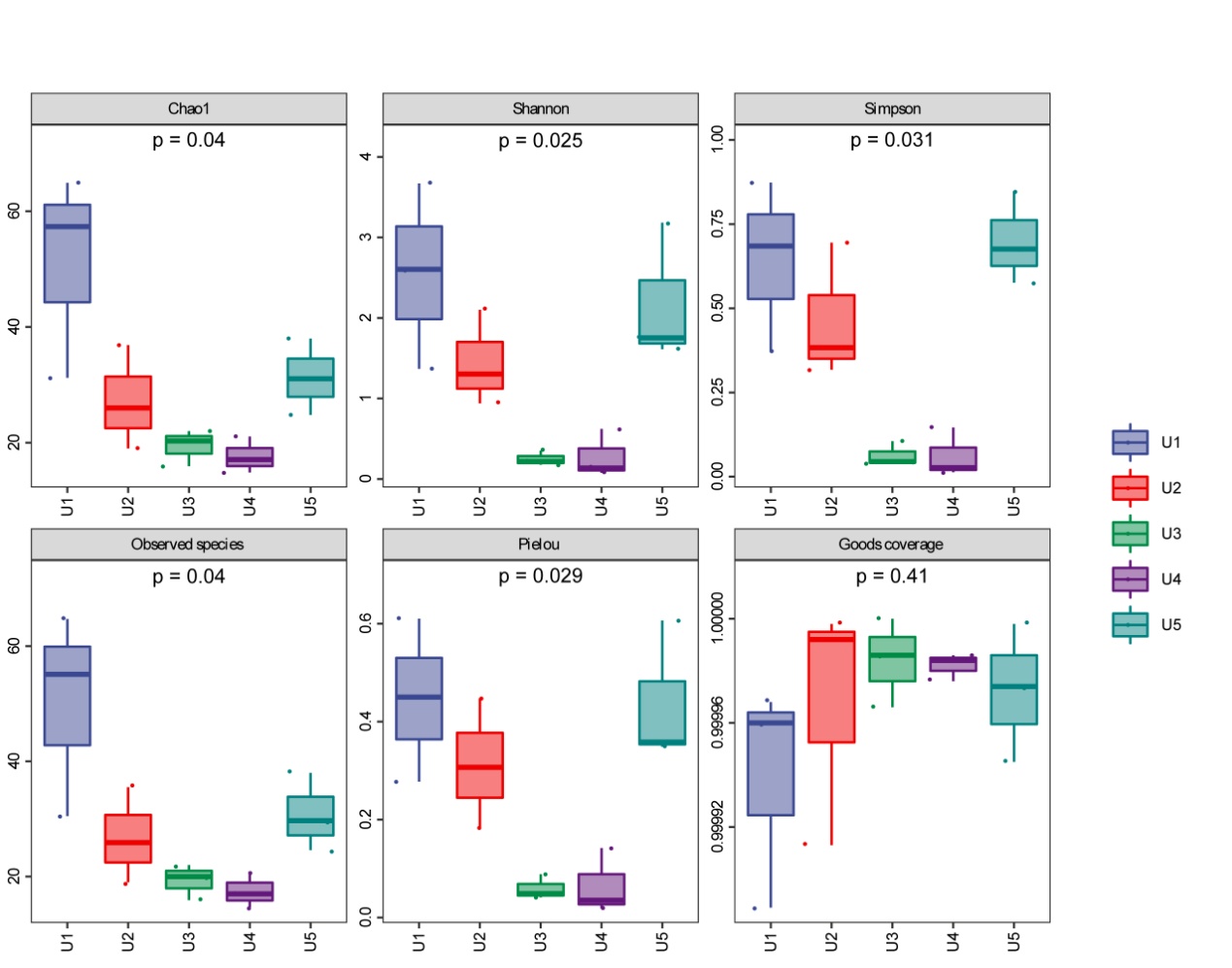


**Supplement Fig. S2.** Alpha diversity of fungal communities associated with *Usnea longissima* based on the results obtained by the Illumina sequencing.

**
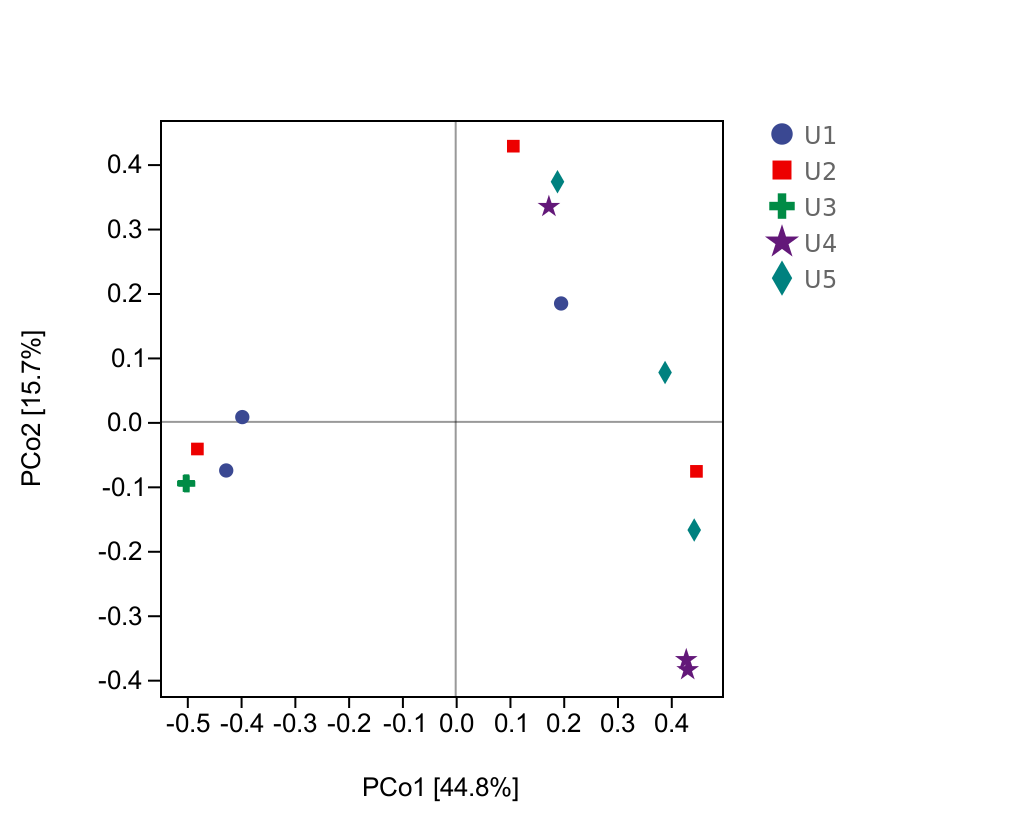
**

**Supplement Fig. S3.** The PCoA of endophytic fungi associated with *Usnea longissima* at different altitudes by Illumina sequencing.


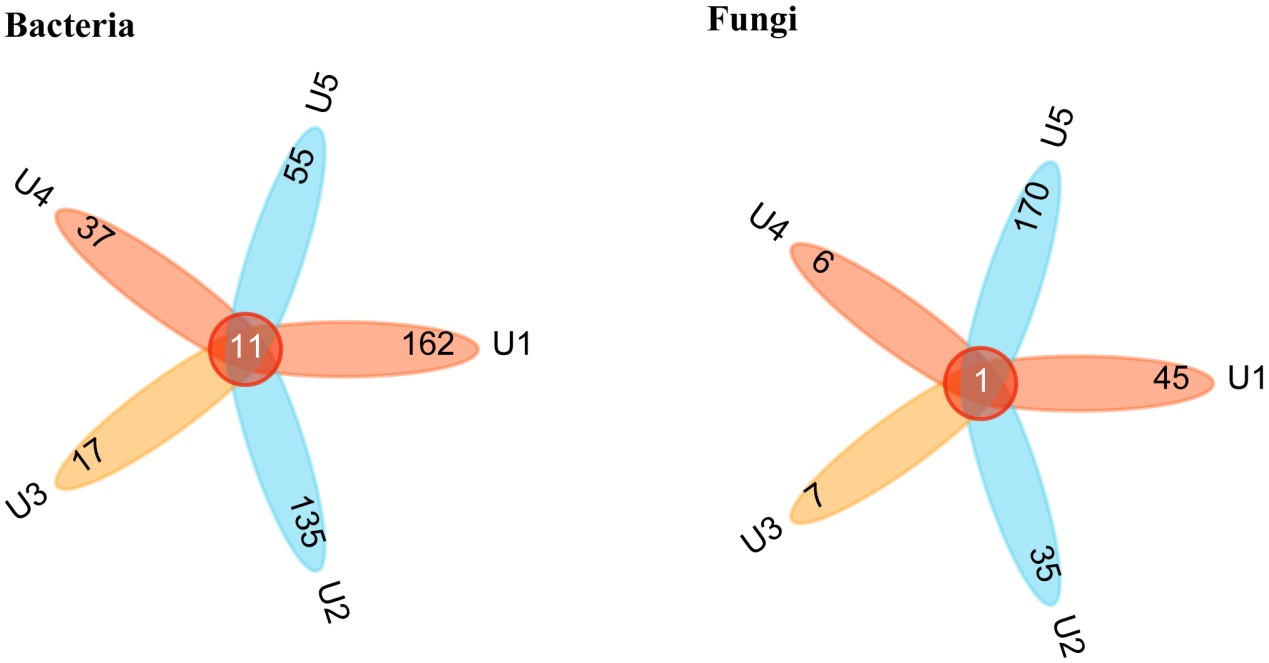


**Supplement Fig. S4.** The shared ASVs of Bacterial and fungal microbial communities associated with *Usnea longissima*.


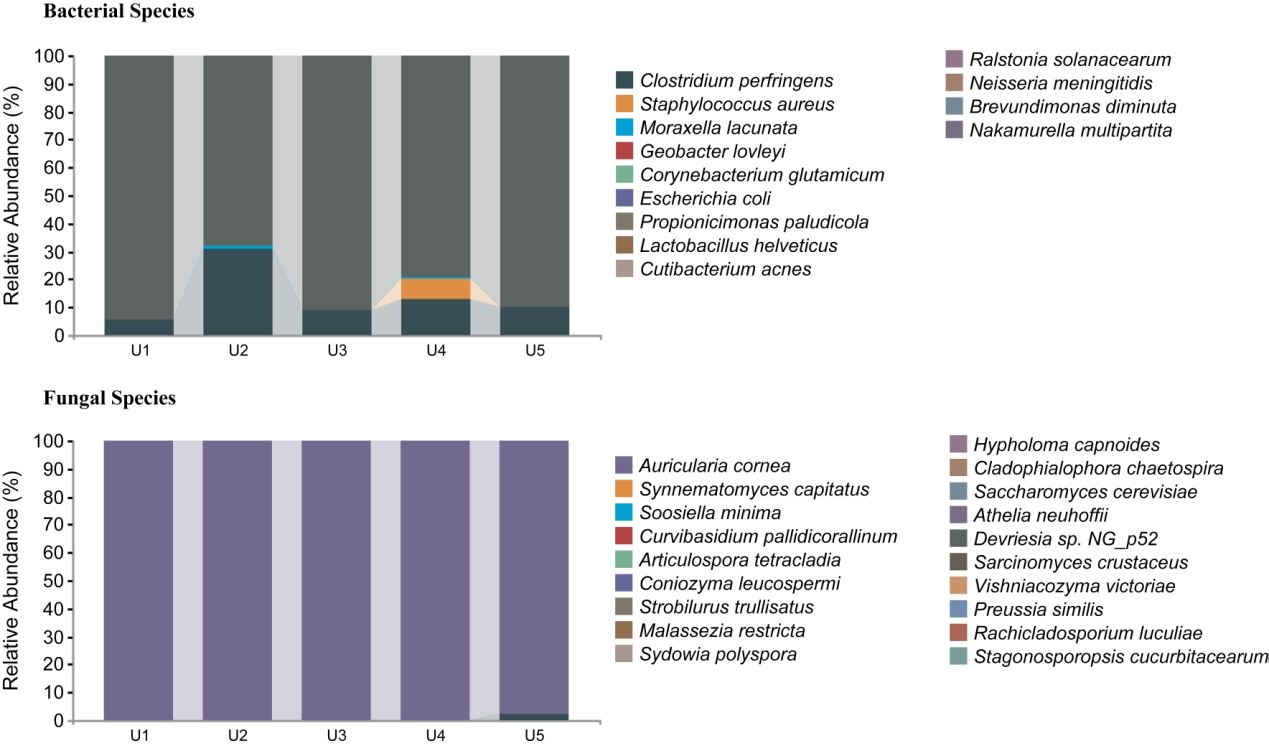


**Supplement Fig. S5.** The relative abundance is distributed at the species level.

.


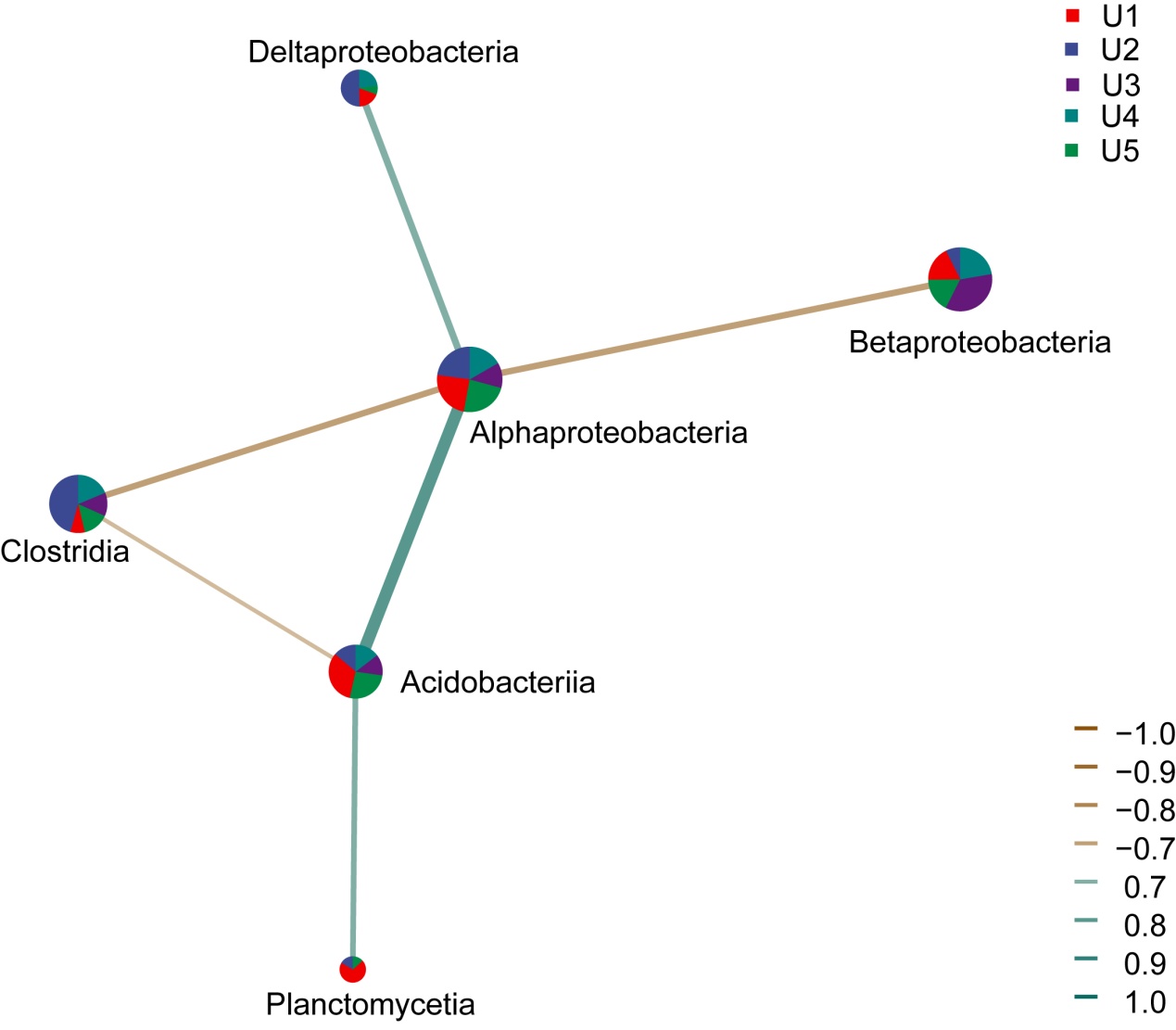


**Supplement Fig. S6.** Co-occurrence network analysis of [bacterial](javascript:;) [community](javascript:;) at the class level.
